# Work More, *Then* Feel More: The Influence of Effort on Affective Predictions

**DOI:** 10.1371/journal.pone.0101512

**Published:** 2014-07-16

**Authors:** Gabriela M. Jiga-Boy, Claudia Toma, Olivier Corneille

**Affiliations:** 1 Department of Psychology, Swansea University, Swansea, United Kingdom; 2 Department of Social Psychology, Tilburg University, Tilburg, The Netherlands; 3 Department of Psychology, Catholic University of Louvain, Louvain-la-Neuve, Belgium; Inserm, France

## Abstract

Two studies examined how effort invested in a task shapes the affective predictions related to potential success in that task, and the mechanism underlying this relationship. In Study 1, PhD students awaiting an editorial decision about a submitted manuscript estimated the effort they had invested in preparing that manuscript for submission and how happy they would feel if it were accepted. Subjective estimates of effort were positively related to participants' anticipated happiness, an effect mediated by the higher perceived quality of one's work. In other words, the more effort one though having invested, the happier one expected to feel if it were accepted, because one expected a higher quality manuscript. We replicated this effect and its underlying mediation in Study 2, this time using an experimental manipulation of effort in the context of creating an advertising slogan. Study 2 further showed that participants mistakenly thought their extra efforts invested in the task had improved the quality of their work, while independent judges had found no objective differences in quality between the outcomes of the high- and low-effort groups. We discuss the implications of the relationship between effort and anticipated emotions and the conditions under which such relationship might be functional.

## Introduction

When we invest effort into something, most of us expect material or emotional pay-offs. A long and thorough process of preparing a journal article, for example, needs a true commitment to research and, more often than not, some anticipated positive emotions should that manuscript prevail the peer-review process. But how do we predict our reactions to potential success? Until recently, affective forecasts were commonly related to people's *neglect* of various important cues - e.g., their psychological immune system, their past affective predictions, their representative memories, or the temporal location of an event. Little is known, however, about the cues people *do* rely upon when making affective predictions.

This research examines whether *effort,* or *task investment*, serves as a cue when people predict their actions' hedonic consequences. We predicted that more effort invested in a task would trigger more positive anticipated emotions, should the task succeed. We further predicted that this effect would be due to people's heuristic belief that their effortful actions lead to better outcomes (“effort heuristic”). We are thus investigating a link between action cues (effort) and affective predictions in an attempt to offer a new insight into the causes of affective forecasting biases.

### Affective predictions: A short summary

An extensive literature has investigated individuals' affective forecasting biases [1]. For example, people tend to overestimate the impact of future events on their feelings (the impact bias, [2]; or the intensity bias, [3], [4]) and expect to feel more enduring emotions than the ones actually experienced (the durability bias, [5]). These inaccurate judgments are due to multiple causes, such as people's tendency to think exclusively about the focal event while failing to consider the consequences of other future events (focalism, [6]), or to imagine the event as more powerful than it actually is (misconstrual, [7]) and to ignore their ability to alleviate the subjective experience of a negative affect (immune neglect, [3], [8]).

A small - but compelling - set of data has further documented the way one's actions are founded on the anticipated emotional consequences of future events [9]. The enjoyment derived from one's current actions or anticipated from one's future experiences seem to play an important role in the way Western individuals, at least, decide about engaging or not in these activities. Decisions such as to become a psychologist vs. a business partner, to marry vs. to divorce someone, to have a challenging vs. a relaxing holiday are largely based on the prediction that one alternative will be more (emotionally) rewarding than the other. In part, this happens because anticipated emotions help us brace for the worst or motivate us to initiate or to persist in goal achievement even when faced with adverse conditions [2], [8], [10]–[13].

This body of research shows that anticipated emotions help sustain effortful actions. In other words, affective forecasts may shape task investment. What we don't know much about, however, is the reverse relationship: Does task investment shape affective predictions? If so, why? We believe this topic is worth investigating because the initial effort people are putting into a task might determine their affective predictions, which in turn could modulate their motivation to sustain that task until the goal is achieved.

### Effort and affective predictions

Effort is rooted in people's everyday life. We tend to expect rewards to be proportional to our investment. Illustrating this view, the “fair wage-effort hypothesis” and equity theory denote that “the ratio of the perceived value of the “inputs” to the perceived value of the “outcomes” would be equal” ([14], pp. 257; [15]). We also tend to value effort and achievement through effort and to experience more negative emotions in case of failure that follows more effort [16]. As a consequence, we tend to reward more the success and punish less the failure of those who we believe have invested a lot of effort into a piece of work [17].

The same should be true when it comes to our own performance and feelings. This is because we face events differing in the amount of effort they require and this may create various expectations about our affective reactions to them. In social psychology, how effort influences judgment is a classic inquiry. Effort influences the extent to which people feel overconfident about realizing a task [13] or the extent to which they engage is self-licensing and hedonic consumption [18]. Work on dissonance and self-perception demonstrated long time ago that effort is positively associated with evaluations of the outcomes of that effort. For example, Aronson and Mills [19] showed that people's liking of a target increased following an unpleasant or effortful experience with that target. The inconsistency between one's effort and one's goal creates a state of dissonance that is reduced by inflating the value of the outcomes of that effort. In line with this idea, some authors suggested that effort inflates affective predictions to better cope with the fact that one has invested a lot of energy in that task [20].

We believe that effort may be an important cue people use when making affective predictions. For example, people may anticipate more intense emotions of gratitude or disappointment, for example, if they perform door-to-door fundraising than online fundraising, because the former is more effortful than the latter. Nevertheless, people also differ in their willingness to invest effort, or in their conscientiousness of fulfilling their tasks, so some of us may spend a lot of time and effort in realizing their actions, while others will only do the minimum to attain the same goal. But what mechanism can explain the impact of effort on affective predictions?

A recent, parsimonious extension of the effort justification thesis stated that effort enhances the value of outcomes simply because it is used as a *cue for quality* (the *‘effort heuristic’*, [21]). In three experiments, these authors reported that people provide higher ratings of quality, value and liking for an outcome (e.g., a poem, a painting, armors) they have thought it had required more effort to produce. The important aspect of the ‘effort heuristic’ hypothesis is that people seem to rely on effort because effort is a generally reliable indicator of quality. Yet, the association between effort and value is imperfect, so that the use of this heuristic can occasionally leads to errors [22]. Our contention is that increased efforts will generally boost up the anticipation of positive emotions *because* people will expect positive results from their work (thus equating effort with quality) and hence expect to feel good about them. This is in line with decision affect theory [23], which shows that utilities and expectations influence the predicted hedonic consequences of our decisions. For example, when people expect positive outcomes, they also tend to show more positive affective reactions.

Therefore, we predicted (1) that more effort invested in a task should result in more positive anticipated emotions (in case of anticipated success), and (2) that this effect would be mediated by quality assessments. Effort may influence people's affective predictions because it can give them an idea about how good they should expect their outcomes to be. In turn, this expectation should trigger feelings, such as happiness if one succeeds, or lack of it, if one fails. Our first research objective is to identify whether people rely on their subjective effort estimates to predict their future affective reactions. Our second objective is to test whether Kruger et al (2004)'s ‘effort heuristic’ mechanism can be one possible explanation of why one would rely on his/her effort to predict his/her happiness. Thus, in addition to Kruger et al.'s [21] who investigated the impact of effort on *other people*'*s performance*, we aim to show that the ‘effort heuristic’ might also concern *one*'*s own performance*.

### Overview of Current Research

The hypotheses above were tested in two studies, one with a correlational (Study 1) and one with an experimental design (Study 2). In Study 1, PhD students awaiting an editorial decision for a submitted manuscript estimated the effort they had invested in preparing that manuscript and how happy they would be if it were accepted for publication. In Study 2, participants produced an advertising slogan in high (vs. low) effort experimental conditions and then predicted their affective reactions if their slogan won an advertising contest. Study 2 also included an objective measure of outcome quality and contrasted it to participants' subjective estimates about the quality of their work.

### Ethics Statement

Both Study 1 and Study 2 have received approval from Swansea University's Department of Psychology' Research Ethics Committee prior to any data collection. Study 1 data were collected online, using SurveyMonkey. For this study, consent form was obtained by asking participants to click “yes” if they agreed with all of the following statements: (1) I confirm that I have read and understood the information above; (2) I understand that my participation is voluntary and that, prior to completion of the study questions, I am free to withdraw at any time, without having to give a reason; (3) I confirm that I am over 18 years of age; (4) I agree to take part in the research. Those who disagreed with one or more of the previous statements were instructed to click “no” and they exited the survey. Study 2 data were collected in public places, and participants signed a written consent form prior to taking part. Their signed consent forms were not linked to participants' data in any way and were stored separately from the data.

In both Study 1 and 2 we collected data referring to participants' age and gender in order to characterize the participant sample. However, as we did not have any a priori hypotheses about age and/or gender roles within our predictions, these will not be part of the analyses reported in the Results sections of Study 1 and Study 2.

## Study 1

We first sought to establish whether there is a positive relationship between the amount of effort one has invested in a task (e.g., preparing a scientific article for submission in a peer-reviewed journal) and the affective prediction regarding that task (e.g., how happy would one be if that article were accepted). We also tested whether the perceived quality of the article mediated the relationship between effort and affective prediction.

### Methods

#### Participants

Initially, 330 students from several European universities took part in this online experiment on a voluntary basis. Of those, we retained data from 139 students (72 women, aged *M* = 27.45 years, *SD*  = 4.62) based on two criteria: That participants were enrolled in a PhD program at that time and that they had already submitted a scientific article for publication in a peer-reviewed journal in their field.

#### Materials and Procedure

Participants were invited to take part in a study about “work-related issues, affect and life satisfaction during PhD”, that took about 15 minutes to complete and was administered online using SurveyMonkey. The study advertisement targeted postgraduate students enrolled in a PhD program, regardless of their research field.

Participants first answered background questions (age, gender, whether they were currently preparing a PhD, the field of study) and a question measuring current happiness (“How happy would you say you are these days?” 1 =  *not happy* to 7 =  *extremely happy*). They then answered questions about their submitted articles (“Have you already submitted an article to a scientific journal?; “When have you submitted your last article?”). Next, participants were instructed to think about their last submitted manuscript and to indicate: “How much would you say you have worked on this article before you have submitted it?”; “How much effort would you say you have put into preparing this article for submission?”, and “How much time have you invested in preparing this article for submission?” (Three items measuring *effort*: 1 =  *very little*, 7 =  *a lot*). Further, we measured the potential mediator, the subjective quality of the article (adapted from Kruger et al., 2004): “How much would you say this article is original?”; “How much would you say this article is complex?”, and “How much would you say this article is well-written?” (for all three, 1 =  *not at all,* 7 =  *extremely*). Participants indicated whether they were the first author and the only author of the paper, when they expected the editorial decision for, and how much scientific experience they had (“Have you already presented your research at conferences? - If yes, for how many times?” and “Have you already published scientific articles in peer-reviewed journals? - If yes, how many?”). Finally, we measured the affective prediction (adapted from [8]) with “How happy do you think you would be if your article were accepted by the journal where you've submitted it?” *(*1 =  *not happy,* 7 =  *extremely happy)*.

### Results

#### Impact of effort on affective predictions

We created a composite score for the ‘effort’ measure by averaging the three items used to measure it (*M* = 5.79, *SD*  = 1.16, α = 0.89). Analyses were done using linear regressions. As predicted, the more effort one had invested in preparing the manuscript, the happier one thought s/he would be if the manuscript were accepted, ***b*** = .17, *SE*  = .08, *t*(137) = 2.11, *p*<.036, η*_p_* ^2^  = .03. This relationship did not depend on current happiness (the effect remained significant, *p*<.028, η*_p_* ^2^  = .03), on the time elapsed since the submission (*p*<.03, η*_p_* ^2^ = .04), on the time remaining until the editorial decision (*p*<.02, η*_p_* ^2^ = .05), neither on whether the participant was the first or the only author of the manuscript (*p*<.04, η*_p_* ^2^ = .03 in both cases).

We have also tested the exploratory research question that the relationship between effort and affective prediction is qualitatively different for people more experienced with the respective task (i.e., publishing manuscripts) than people less experienced with that task. We created a composite score for ‘experience’ by averaging the scores at the two items measuring it (“Have you already presented your research at conferences? - If yes, for how many times?” and “Have you already published scientific articles in peer-reviewed journals? - If yes, how many?”), *r*(136) = .27, *p*<.01. Most participants (84 out of 139) were completely inexperienced in publishing manuscripts (their mean experience was zero). For those inexperienced participants, the relationship between effort and expected happiness approached significance, *r*(84) = .20, *p* = .07. For the more experienced participants (with a mean experience superior to 3.5), however, the results indicated that effort was no longer related to expected happiness, *r*(55) = .14, *p* = .31.

#### The ‘effort heuristic’ mechanism

**Figure 1:**
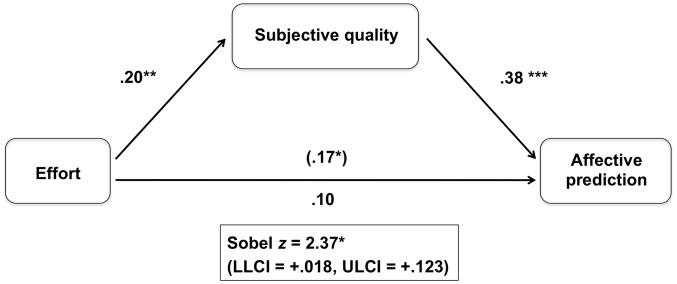
Mediation of the Effort - Affective Prediction (expected happiness) Relationship by Subjective Quality (Study 1). Values represent unstandardized regression coefficients. The coefficient in parentheses represents the association between Effort and Affective Prediction when Subjective Quality is not included in the model (* *p*<.05; ** *p*<.01; *** *p*<.001).

We expected that the effort invested in a task (the manuscript) would predict the expected happiness because people associate higher effort with higher quality outcomes. To test this mediation hypothesis, we first created a composite score for the ‘subjective quality of the article’ by averaging the three items used to measure it (*M* = 4.81, *SD*  = 1.08, α = 0.70). Effort predicted subjective quality of the article, ***b*** = .20, *SE* = .08, *t*(137) = 2.54, *p*<.01, η*_p_* ^2^  = .04, indicating that the more effort one invested in preparing the manuscript, the better quality one thought the manuscript would be. Second, we tested whether subjective quality of the article predicted expected happiness (controlling for effort), which was confirmed, *b* = .38, *SE*  = .08, *t*(136) = 4.44, *p*<.001, η*_p_* ^2^  = .12: The better quality one estimated the article was, the happier one expected to feel if it were accepted. And finally, when controlling for the influence of the subjective quality of the article, effort no longer predicted expected happiness, ***b*** = .10, *SE* = .08, *t*(136) = 1.26, ***p*** = .21, η*_p_* ^2^ = .01, Sobel *z* = 2.37, *p*<.05 (Figure 1).

We have also investigated the mediation hypothesis using bootstrapping analyses with 1000 samples [24], which revealed a significant indirect effect of effort on expected happiness through subjective quality of the article (LLCI = +.018, ULCI = +.123).

### Discussion

Study 1 results confirmed our hypothesis about a positive relationship between effort and affective predictions: The more effort one declared having invested in an article, the happier one predicted s/he would feel, if the article were accepted. We further predicted that this affective forecasting effect would be mediated by the subjective quality of the article. This prediction was also supported: PhD students who estimated having invested relatively more effort in preparing a manuscript thought their effort had led to a higher-quality article, which led them to expect feeling happier in case of success. This study is the first demonstration of an effort-based affective forecasting effect and of the meditational role of an effort heuristic.

One plausible alternative explanation for Study 1 results could be the fact that if participants think their manuscript is good, they would probably sent it to a good-quality academic journal. Therefore, acceptance would have an objectively higher value for these compared to those who sent their manuscript to a less good-quality academic journal. We have no empirical data that would allow us to examine this alternative account regarding Study 1 results. However, in Study 2, we attempt to demonstrate that the relationship between effort and future affective predictions depends on the effort heuristic, this time using an experimental design and materials that are devoid of Study 1 limitations discussed hereby.

## Study 2

Study 2 was aimed at providing direct *experimental* evidence for our hypothesis. We predicted that participants randomly assigned to a high-effort condition would expect to feel happier if the task they engaged in (creating an advertising slogan) had succeeded than participants assigned to a low-effort condition. Again, we expected this affective forecasting effect to be mediated by the subjective quality of the task. A second objective was to test the notion that the ‘effort heuristic’ strategy can lead participants to overestimate the quality of their outcome. Specifically, we expected that although participants in the high-effort condition would perceive their slogan as qualitatively better than participants in the low-effort condition, no *objective* difference in quality between these two conditions would actually be observed. This prediction, if supported, would complement Krueger et al.'s [21] research in showing that effort can become a cue for quality not only when evaluating others' work, but also when evaluating one's own outcomes.

### Method

#### Participants

Thirty-five students (17 women, aged *M* = 22.89 years, *SD*  = 3.64) took part in this lab-based experiment on a voluntary basis.

#### Materials and Procedure

Participants were invited to participate in “a study on advertising and creativity”. They were given a scenario according to which a well-known national advertising agency was launching a students' contest, in which participants could win a monetary prize and the opportunity to do an internship in advertising within the agency. In order to win, participants needed to create an advertising slogan for a product supposedly included in the agency's portfolio (an energy drink). They were provided with the main features of the product (e.g., ingredients, caloric intake) and received instructions about how to create the slogan, which represented the effort manipulation. Participants in the *high-effort condition* were told to take all the time they needed to think of an advertising slogan because “the ability to generate ideas carefully is seen as crucial in order to work in this agency”. They were also instructed not to settle for the first slogan that came to mind, but to generate as many slogans as possible and to choose one of them only when they finished. Those in the *low-effort condition* were told to think of a slogan as quickly as possible, because “the ability to generate ideas rapidly is seen as crucial in order to work in this agency”. They were also instructed to settle for the first slogan that came to mind.

Upon completion of this task, participants answered questions measuring the variables of interest: Subjective quality of the slogan they created (“How much do you believe the slogan you have created is interesting/good/original/attractive?” 1 =  *not at all*, 7 =  *extremely*) and predicted happiness (“How happy do you think you would be if your slogan won the contest?” 1 =  *not happy;* 7 =  *extremely happy*, adapted from [8]). We also measured perceived effort (“How much effort did you invest in creating your slogan?”; “How much did you think before finding your slogan?”, 1 =  *very little*, 7 =  *a lot*) and perceived difficulty of the task (“How difficult did you find this task?” 1 =  *not at all*, 7 =  *very difficult*).

### Results

#### Manipulation checks

We created a composite score for ‘perceived effort’ by averaging the scores of the two items measuring it, *r*(34) = .79, *p*<.001. Participants in the high-effort condition estimated they had invested more effort in creating the slogan (*M* = 3.78, *SD*  = 1.23) than participants in the low-effort condition (*M* = 2.47, *SD*  = 1.27), *F*(1, 33) = 9.60, *p*<01, η*_p_* ^2^  = .22. As the high-effort (*M* = 3.50, *SD*  = 1.10) and the low-effort conditions (*M* = 3.06, *SD* = 1.98) did not differ in terms of perceived difficulty (*F*<1), we excluded the possibility that the effort manipulation had also introduced variations in perceived difficulty of the task.

#### Impact of effort on affective predictions

As predicted, ANOVAs revealed that participants in the high-effort condition thought they would be happier if their slogan won the advertising contest (*M* = 6.28, *SD* = 0.96) than those in the low-effort condition (*M* = 4.76, *SD* = .30), *F*(1, 33) = 6.56, *p*<01, η*_p_* ^2^  = .16. This effect remained significant when ‘current happiness’ was entered as covariate, *F*(1, 33) = 6.62, *p*<01, η*_p_* ^2^  = .17.

#### The ‘Effort heuristic’ mechanism

**Figure 2:**
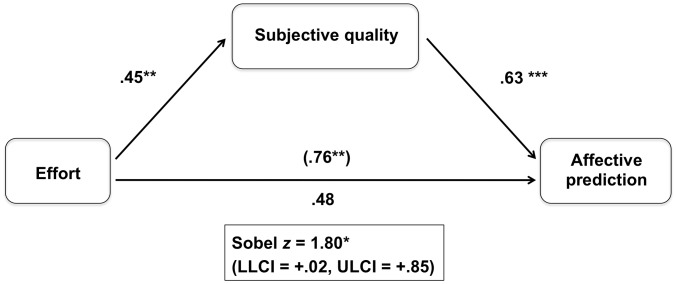
Mediation of the Effort - Affective Prediction (expected happiness) Relationship by Subjective Quality (Study 2). Values represent unstandardized regression coefficients. The coefficient in parentheses represents the association between Effort and Affective Prediction when Subjective Quality is not included in the model (* *p*<.07; ** *p*<.05; *** *p*<.01).

We created a composite score for the ‘subjective quality of the slogan’, by averaging the four items used to measure it (*M* = 4.41, *SD*  = 1.34, α = 0.92). To test the mediation hypothesis, we used linear regressions and we first tested whether the high-effort condition resulted in high subjective quality ratings of the slogan. This was indeed the case, ***b*** = .45, *SE*  = .22, *t*(33) = 2.06, *p*<.05, η*_p_* ^2^  = 11. Second, we tested whether higher subjective quality predicted higher anticipated happiness (controlling for effort), which was also confirmed, *b* = .63, *SE*  = .21, *t*(32) = 2.94, *p*<.01, η*_p_* ^2^  = .18. And finally, when controlling for the influence of subjective quality of the slogan, effort no longer predicted anticipated happiness, ***b*** = .48, *SE*  = .28, *t*(32) = 1.68, ***p*** = .10, η*_p_* ^2^  = 05, Sobel *z* = 1.80, *p* = .07 (compared to the direct effect of effort on anticipated happiness, *b*  = .76, *SE* = 29, *p*<.05, η*_p_* ^2^  = 16, see Figure 2). Bootstrapping analyses with 1000 samples [24] revealed a significant indirect effect of effort on expected happiness through subjective quality of the article (LLCI = +.02, ULCI = +.85).

#### Subjective vs. objective quality of the slogan

Participants in the high-effort condition estimated their slogan was qualitatively better (*M* = 4.84, *SD*  = 1.06) than participants in the low-effort condition (*M* = 3.95, *SD*  = 1.48), *F*(1, 33) = 4.25, *p*<.05, η*_p_* ^2^  = .11. We were interested in whether this difference between high- and low-effort conditions reflected a biased evaluation (the ‘effort heuristic’) or rather an objective difference in the quality of the slogan. Thus, we asked 24 independent judges (14 women, aged *M* = 21.03 years, *SD*  = 3.51) to evaluate the quality of the slogans. All judges were presented with the 35 slogans generated by participants and were asked to rate them on several dimensions: “How much do you believe this advertising slogan is interesting/good/original/attractive?” (1 =  *not at all*, 7 =  *extremely*). Next, we created two scores for the objective quality of the slogan, one for those created in the high-effort condition (α = 0.94) and one for those created in the low-effort condition (α = 0.95). The former were *not* judged as qualitatively better (*M* = 2.54, *SD*  = 0.52) than the latter (*M* = 2.48, *SD*  = 0.58), *F*(1, 23) = 1.02, *p* = 32, η*_p_* ^2^  = .04, which confirmed that effort inflated the subjective evaluations of the slogan quality instead of leading to real, objective differences in outcome quality.

Using an experimental design, Study 2 confirmed that additional effort invested in a task elicits the feeling that one's work resulted in better outcomes, while not producing objectively different outcomes. This subjective quality feeling, in turn, increased one's anticipated happiness in case of success.

## General Discussion

In two studies we investigated whether effort invested in a task increases one's anticipated happiness if the task was successful. Study 1 showed that the more effort a PhD student had invested in preparing a manuscript for submission to a peer-reviewed journal, the happier s/he predicts s/he would feel if the manuscript were accepted for publication. Furthermore, Study 1 suggested that a potential explanation for this relationship was the subjective quality of the task: The more effort one estimates having invested in a task, the more one expected the task outcome to be good, which in turn increased anticipated happiness. Study 2 tested the same hypotheses, this time using an experimental manipulation of effort in the context of a different task: When working in high (vs. low)-effort conditions, participants estimated they had produced better outcomes, which in turn increased their predicted happiness. Importantly, no objective difference in the quality of the task outcomes accounted for this latter result. That is, participants subjectively thought that the extra efforts they had engaged in the task improved the quality of their work, while independent judges had not observed any objective differences in quality between the two conditions. Hence, effort inflated participants' affective prediction through overestimations of their work quality.

How can this effect be explained? According to the ‘effort heuristic’ model, effort enhances the subjective value of outcomes because it functions as a cue for estimating the quality of one's work [21]. Consequently, if people estimate their work to be better, they may also expect positive affective consequences for that work (one would generally prefer to have a good, rather than an average manuscript accepted for publication). However, our results go beyond the initial evidence for the ‘effort heuristic’ because a) they *appeal* to ‘effort heuristic’ to *explain* a relationship between current effort and future affective predictions that was not previously documented and b) they go beyond the current ‘effort heuristic’ literature by showing that it occurs regarding effort one has performed himself/herself, instead of someone else.

Effort heuristic is a parsimonious extension of the classic “effort justification” account, according to which the effort one invests in a task may carry out the meaning that the task is “more interesting” [19] in order to justify the cost of pursuing an effortful goal. In other words, one would need to have invested high effort into something in order to derive pleasure, likeness or happiness, because this is part of the justification process. According to the effort justification hypothesis [19] people might have logical associations or naïve scripts linking effort to quality and anticipated happiness.

One could argue that our studies might have reinforced this script because of the order in which the concepts were measured. In Study 1 the causality of effort in determining the quality and, subsequently, the predicted happiness could have been inferred by the item order. In Study 2, where effort was manipulated, the order was again consistent with the causal script. But recent data collected in our laboratory refute this possibility: when participants were asked to imagine the causal chain (people imagined their effort, the predicted quality and affect) they expected higher quality after high effort (which is consistent with the effort heuristic), but they did not expect higher happiness. This suggests that the causality of the effort cannot simply be inferred by order, as people need to have experienced the effort in order to predict their affect.

Whether effort is a simple heuristic that allows fast judgments about one's expected feelings, a shutter button for justification processes or part of a naïve script is a debate that cannot be solved by our results alone. Instead, we point to an element so far ignored in the affective forecasting literature (e.g., [25]): Namely, what people *do focus on* when they predict their future emotions. We suggest that the effort invested in a task matters when forming affective forecasts, regardless of how accurate these forecasts might be. Study 2 indeed suggests that the relationship between effort and affective predictions is explained by one's exaggerated perception of the quality of one's work, while there was no objective difference in quality for works produced under high or low effort conditions. We thus believe that the most interesting question to be asked with regard to the impact of effort on affective predictions is whether this relationship is *functional* or not, and in what conditions one uses effort cues to estimate an emotional future reward.

### Functionality vs. perils of anticipated emotions shaped by effort

How important are anticipated emotions for motivating current behavior? This question has long been at the heart of motivation and self-regulation research (e.g., [12]). The possibility that the effort-affective predictions relationship is a motivated or functional process is worth discussing. People may anticipate emotional rewards from their actions because they estimate them as qualitatively better, therefore expecting the best results to meet their efforts. Effort may help people exaggerate the positive consequences of their actions in terms of outcomes (success) and affect (happiness) to motivate themselves to pursue with one action. Moreover, because effort is a controllable aspect of behavior, people should be more confident that positive consequences would derive from effortful actions, which in turn may motivate them to invest further efforts in these actions. For this reason perceived effort should matter more than real effort and should speak to the functionality of the relation described here. If the same task is perceived as effortful by Person A and effortless by Person B, only Person A will expect higher happiness in case of success because this would motivate her to get involved in that task. Recent literature (e.g., [26], [27]) outlines precisely how *subjective* judgments of temporal distance or emotions take precedence in determining one's motivation for future tasks or one's judgments of present tasks.

One might also consider how legitimate and normative it is that more effort brings more happiness. This social justice explanation [28] cannot be, at present, accounted for in our results, but future research might find interesting to specify the conditions in which more effort is associated with more reward. For example, some [16] suggested that the effort-reward relationship is stronger the less one has to contribute himself/herself to that task (e.g., a teacher might expect the best school results with more effort, but a pupil might expect the best results with little effort).

Effort seems to serve as a motivator because it inflates our expectation to derive pleasure from our actions. However, this possibility cannot ignore the important debate between pros (e.g., [29]) and cons of positive illusions and motivation. People prone to self-enhancing biases progressively show disengagement from academic tasks (e.g., decline in valuing their academic grades over the years) and lower self-esteem and well being [30]. Because effort can create false expectations about how good one has performed or about how good one will feel when achieving future goals, it may trigger disappointment if outcomes are unsuccessful or disengagement from goal pursuit when one realizes his/her predictions were wrong.

A last detail that is worth mentioning regards the finding reported in the footnote. With additional analyses, we found that among Study 1 participants, 60% were completely inexperienced in publishing manuscripts and 40% were somewhat experienced. By testing the relationship between effort and predicted happiness separately for each of these sub-samples, we found that it approached significance for those inexperienced, but not for those somewhat experienced. This could indicate that both Studies 1 and 2 included a majority of inexperienced participants, both in terms of publishing manuscripts or more creative tasks such as creating a slogan. Thus, effort is related to affective prediction only for people unfamiliar or inexperienced with the respective task. We believe this possibility regarding the role of experience or expertise is worth exploring in future research, but in the current research we have decided to keep it as a footnote due to the post-hoc nature of this analysis.

### Limits and further research

The measures of predicted happiness used here were not followed by measures of “real” happiness, as most affective forecasting research does. However, in light of recent meta-analyses [1], we know that people are generally inaccurate in an absolute sense (i.e., they all expect to feel worse than they actually will), but in a more relative sense they seem to be quite accurate about their future feelings. Regarding our results, this means that participants who declared having invested more effort and who expected to feel the happiest will, eventually, feel happier than others in case of success (but perhaps more disappointed in case of failure).

Another limitation is that participants were only asked to anticipate positive affect. However, we predict that effort would shape anticipated negative affect in the same way. This is because effort has an impact on the intensity of the affective response, and not on the type or polarity of affective response. Research on affective forecasting showed that people exhibit the same bias (overestimate the intensity of their affect) regardless of whether the event is positive (in which case people overestimate happiness) or negative (in which case people overestimate sadness, e.g., [31]).

Related to the previous point, one could argue that the focus on effort might have led participants to frame their affective forecast in terms of not losing, instead of winning. This could occur because rejection can be more painful when one has put much effort into a manuscript or a slogan. Consequently, participants might have predicted their anticipated relief from not being rejected rather than from their anticipated happiness. Research on motivational states (e.g., regulatory focus, [32]) does indeed predict differences in various aspects of goal pursuit, such as one's affective responses that accompany goal pursuit. Measuring people's motivational orientation (promotion, prevention) or directly framing the situation as success seeking or failure avoidance might moderate the type of affective response. However, the relation between effort and the intensity of the affect (which is of interest here) should be the same, regardless of the polarity of the affective response (positive or negative).

The positive relationship between effort and predicted happiness suggests that effort could induce other types of judgment biases as well, such as durability, focalism, or misconstruction. For example, effort may accentuate people's propensity to focus on the task or the event requiring the effort at the expense of other tasks, when predicting their affective reactions [6]. The effort invested in a future event also influences the perception of temporal distance to that event, so that effortful events are perceived as happening closer in time that effortless ones [26]. Moreover, temporally close events seem to induce more intense emotions than temporally distant events [27]. Taken together, these studies suggest that more effort invested in a task may induce more intense emotions because effortful events are perceived as temporally closer. An interesting question is whether effort may have a similar impact on the affective predictions in the case of *negative* emotions. A research [33] showed that effort influences the intensity of regret and disappointment. It would be provocative to show that effort is also related to anticipated negative emotions. For example, effort may make people aware of their defensive mechanisms, thus reducing the immune neglect bias [34].

### Coda

This research provides original evidence (1) that people rely on effort invested in a task to predict how happy they would feel, if successful. It also reveals that (2) this effort influence on affective predictions is mediated by higher perceived quality of one's work and that (3) the latter assessments may be inflated. This work extends previous research on how *action-related cues* – such as effort, task investment, or the action's current emotional intensity – shape estimations of more volatile parameters, such as future emotions. Effort inflates our affective predictions, which results either in some priceless help for maintaining current actions or initiating future ones, or in increased susceptibility to overestimate the quality of one's work and deception if outcomes are negative.
